# BRAF Testing in Melanoma and Colorectal Cancer in Latin America: Challenges and Opportunities

**DOI:** 10.7759/cureus.31972

**Published:** 2022-11-28

**Authors:** Renata D Peixoto, Jad Joseph Abbas Chakhtoura, José L Aguilar-Ponce, Hernan Garcia-Rivello, Angela M Jansen, Rafael Parra Medina, Allan Ramos-Esquivel, Stephen Doral Stefani

**Affiliations:** 1 Medical Oncology, Centro Paulista de Oncologia, Sao Paulo, BRA; 2 Anatomical Pathology, Hospital Rafael Angel Calderón Guardia, San Jose, CRI; 3 Oncological Center, Médica Sur, Mexico City, MEX; 4 Pathology, Hospital Italiano de Buenos Aires, Buenos Aires, ARG; 5 Pathology, Institute of Translational Medicine and Biomedical Engineering (IMTIB), Buenos Aires, ARG; 6 Genetics, Americas Health Foundation, Laguna Beach, USA; 7 Pathology, Instituto Nacional de Cancerología, Bogota, COL; 8 Caja Costarricense de Seguro Social, Hospital San Juan de Dios, San José, CRI; 9 Health Technology Assessment, Unimed, Porto Alegre, BRA

**Keywords:** recommendations, precision medicine, latin america, treatment, colorectal cancer, melanoma, braf testing

## Abstract

The incidence of colorectal cancer in Argentina and Brazil has reached levels comparable to those in higher-income countries. Similarly, the incidence of melanoma in Latin America has increased during the past decades. *BRAF*mutation is seen frequently in melanomas and colorectal cancer. Discovering the expression of this specific biomarker in both cancers has unleashed the potential for targeted molecular therapies.In patients with *BRAF*-mutated melanoma, adopting a combined targeted treatment approach has shown a dramatic increase in overall survival. However, several barriers impede the development of early *BRAF* testing in Latin America, jeopardizing the potential for personalized therapies and care. To address this, the Americas Health Foundation convened a virtual meeting of Latin American oncologists to address the barriers to *BRAF* testing in melanoma and colorectal cancer. During a three-day conference, expert oncologists used literature reviews and personal experience to detail the barriers to early *BRAF* testing in their region. They proposed actionable steps to overcome the barriers identified, which included deficiencies in knowledge, treatment options, equitable distribution, timely results, and local data on *BRAF* mutations. Oncologists proposed several actions to overcome barriers, including raising public and healthcare awareness about the importance of BRAF testing, expanding treatment options in clinics across the region, developing centers in underserved areas, and increasing affordable treatment options for patients who test positive for BRAF mutations.

## Introduction and background

*BRAF* mutations are found in about 50% of all individuals with melanoma and 10% of those with colorectal cancer (CRC) [1,2]. The discovery of this unique biomarker’s presence in both cancers has opened the door to targeted molecular therapy [3]. Adopting a combined targeted treatment approach in patients with *BRAF*-mutated melanoma has resulted in a dramatic increase in overall survival (OS); patients receiving combination therapy targeting *BRAF* and *MEK* have an OS of 25.9 months, whereas those receiving only chemotherapy have an OS of six months. In patients with *BRAF*-mutated CRC [4], a combination of a *BRAF* inhibitor with an anti-estimated glomerular filtration rate (eGFR) therapy yields a response rate of 48% in the first line [4]. The benefits of precision medicine in improving prognosis and expanding treatment options have become increasingly evident as therapeutic trends evolve.

Personalized treatment and tailored therapy depend on genetic testing. Multiple methods can identify *BRAF* mutations. Sanger sequencing, immunohistochemistry (IHC), pyrosequencing, polymerase chain reaction (PCR), and next-generation sequencing (NGS) are examples. Early and accurate cancer diagnostics are key to treatment success. *BRAF* molecular testing is needed to optimize therapy and prognosis [5].

Melanoma and CRC constitute a significant burden on Latin American populations, governments, and healthcare systems. The incidence of melanoma in Latin America (LA) has increased in recent decades, reflecting global patterns. In 2020, there were 18,881 new cases in LA and the Caribbean, with 5,657 deaths. Incidence rates are much lower in LA than in Europe (150,627 new cases) or Northern America (105,172), possibly due to underdiagnosis [5]. Nonetheless, melanoma in LA surpasses both regions in terms of mortality, with approximately 29% of all cases resulting in death. The divergence in survival rates reveals a critical problem: the existing inadequacies in care for individuals with *BRAF*-mutated melanoma.

CRC is the fifth most common cancer in LA, accounting for 134,943 new cases in 2020. CRC is a heterogeneous disease of high relevance and the third most common cancer worldwide. According to the International Agency for Research on Cancer (IARC), CRC is the third most common neoplasia among individuals over 50 and is projected to increase by 44% by 2030 [6,7]. Globocan 2020 data for LA revealed an incidence and mortality of 134,943 new cases/year and 69,435 deaths/year, respectively. The cumulative incidence risk is nearly 2% for South America and the Caribbean and 1.19% for Central America. The cumulative mortality risk is about 1% for South America and the Caribbean and 0.6% for Central America, considering both men and women. CRC cases in LA are increasing due to demographic changes such as life expectancy increases and dietary pattern modifications, among other factors [7-9]. Its incidence in Argentina and Brazil has reached comparable levels in higher-income countries (HIC) [10].

This narrative review aims to perform a needs assessment of timely molecular testing of the *BRAF *gene in LA and provide recommendations from experts in the field.

## Review

Americas Health Foundation (AHF) conducted a literature review using PubMed, MEDLINE, and EMBASE to identify LA-based scientists and clinicians who have published in oncology, pathology, and *BRAF* tests since 2016. AHF used the following search terms: 'BRAF,' 'BRAFV600', 'molecular testing,' 'melanoma,' and 'colorectal cancer' in combination with 'Latin America' from January 1, 2016 to January 10, 2022. The identified articles were in English, Portuguese, and Spanish. Augmenting this search, AHF contacted opinion leaders from LA's medical field to corroborate that the panelists chosen adequately represented the field. They met on June 13-15, 2022, to develop recommendations for widespread *BRAF* testing for melanoma and CRC in LA.

AHF assigned each panel member a question on *BRAF* testing for early diagnosis and treatment of melanoma and CRC in LA. Individual panel members answered questions based on the AHF literature review, their own reviews, and personal knowledge. The panel reviewed and amended each answer during a three-day meeting and many discussion rounds. Following the meeting, the panel evaluated and approved the final document. After the session, the completed article was given to the panel for assessment and approval.

Guidelines for *BRAF* testing

**Figure 1:**
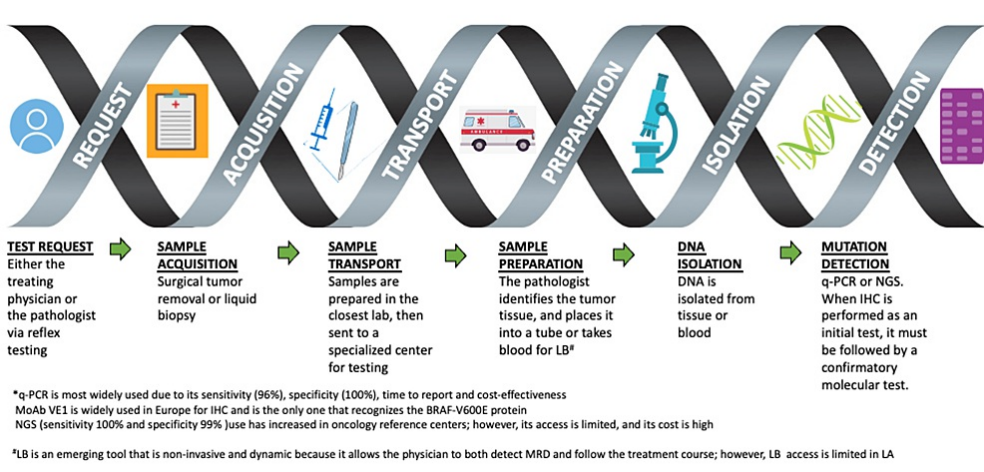
Processes used to detect BRAF mutations The figure depicts the many steps and resources, including personnel, needed to obtain a sample, test for BRAF mutations, and report the results. LB: liquid broth; IHC: immunohistochemistry; q-PCR: quantitative-polymerase chain reaction; NGS: next generation sequencing; MoAB: monoclonal antibody; MRD: measurable residual disease Image credit: Authors

Current guidelines do not recommend a specific technique to detect *BRAF* variants. However, the most widely used is real-time PCR (RT-PCR) due to its sensitivity (96%), specificity (100%), reporting time, and cost-effectiveness. In some centers, IHC is performed as an initial test with subsequent confirmation using techniques such as RT-PCR or NGS [1,5,11]. The monoclonal antibody VE1, the only one that recognizes the *BRAF*-V600E protein, is widely used in Europe [7]. NGS technology has increased in oncology reference centers; however, access to this method is limited in LA [12]. It features a sensitivity of 100% and specificity of 99% for detecting different *BRAF* mutations but results in delayed reporting and high upfront costs [1,5,13]. Liquid biopsy is another emerging tool, although its access is limited in LA [5]. This non-invasive and dynamic tool is advantageous because it allows the physician to detect measurable residual disease (MRD) and follow the response to treatment. *BRAF* testing is a complicated process that involves numerous specialists, laboratories, equipment, and reagents (Figure 1).

Melanoma

Ascierto et al. recommended screening all patients with advanced melanoma (unresectable stage III and stage IV), primarily when metastatic, for the *BRAF*-V600 mutation. Patients at high risk of recurrence (stage IIIB and IIIC) should additionally be screened for mutations [1]. Because *BRAF*-mutant melanomas are aggressive, it is critical to detect whether patients with melanoma have tumors with the *BRAF* mutation as soon as possible to choose the best treatment. The COMBI-AD trial, for example, discovered that 12 months of adjuvant therapy with dabrafenib plus trametinib resulted in a substantial survival increase without relapse of distant metastases in patients with stage III melanoma compared to placebo [14]. Dabrafenib and trametinib are approved for adjuvant therapy in stage III melanoma. Moreover, patients with metastatic, recurrent, or inoperable melanoma harboring a *BRAF* mutation (V600E or V600K) are also candidates for targeted therapy, especially when a rapid response is clinically needed. The latter has been shown in the COMBI-AD trial. Because combination therapy with *BRAF* and *MEK* inhibitors has the potential for significant long-term treatment benefits, identifying a patient's *BRAF* mutation status should be a priority for the clinician. Therefore, in cases where targeted therapy is preferred over immunotherapy, *BRAF* mutational tests are required at diagnosis rather than after first-line progression.

CRC

Detection of *BRAF* mutations in CRC is of high importance due to clinical relevance regarding responsiveness to monoclonal antibody treatment that targets eGFR, a worse outcome in the presence of V600 mutations, and, in some situations, it facilitates the determination of whether the carcinoma is of somatic origin [6,15,16].

Given the poor prognosis associated with the *BRAF*-V600E mutation in CRC, many oncologists consider first-line triple therapy with FOLFOXIRI (folinic acid, 5-fluorouracil, oxaliplatin, and irinotecan) with or without bevacizumab for such patients because a subgroup analysis of the TRIBE study demonstrated the benefit of triple (FOLFOXIRI) versus double (FOLFOX (5-FU, Leucovorin, Oxaliplatin) or FOLFIRI (folinic acid, fluorouracil, and irinotecan)) therapy with bevacizumab [17]. However, not all studies point to the advantage of treatment intensification. For patients with metastatic CRC who meet the study criteria, upfront FOLFOXIRI plus bevacizumab followed by reintroduction of the same regimen after disease progression appears to be a preferable therapeutic strategy to sequential administration of chemotherapy doublets in combination with bevacizumab [18].

Due to the success of *BRAF* inhibitors in other tumors, several studies have evaluated the use of *BRAF* inhibitors with or without *MEK* inhibitors in metastatic CRC (mCRC) but without achieving the same benefits as in melanoma [19-22]. CRC cells depend on eGFR activation as a feedback mechanism in the presence of *BRAF* inhibitors, leading to sustained activation mediated by phosphoinositide 3-kinases (PI3Ks) [23]. This makes combining a *BRAF* inhibitor with an anti-eGFR antibody more effective.

*BRAF* inhibitors were first tested in refractory CRC. In patients with previously treated *BRAF*-V600E, a randomized phase II study showed a median progression-free survival (PFS) gain of 2.4 months with the combination of *BRAF* inhibitor (vemurafenib), irinotecan, and cetuximab [24]. Subsequently, the phase III BEACON trial showed benefits in increasing OS, PFS, and response rate (RR) with the combination of encorafenib (a *BRAF* inhibitor) and cetuximab with or without binimetinib (a *MEK* inhibitor) when compared to irinotecan or FOLFIRI plus cetuximab in previously treated patients. There was no difference in OS with the addition of binimetinib to encorafenib plus cetuximab, so cetuximab and encorafenib became the standard of care in this scenario [25]. More recently, the phase II Anal Cancer/HSIL Outcomes Research (ANCHOR) study showed a response rate of 48% for the combination of cetuximab, binimetinib, and encorafenib in the first-line, but with a relatively short median PFS of 4.9 months [4]. Therefore, there are still doubts about the best first-line treatment option for patients with *BRAF*-mutated mCRC.

For patients with *BRAF*-mutated mCRC who also harbor high microsatellite instability (MSI-H), first-line treatment with pembrolizumab is typically the preferred option because complete responses (CRs) have been reported [26]. Unfortunately, advances in treating *BRAF*-mutated CRC do not apply to most patients in LA, given the lack of access to *BRAF* inhibitors.

Reflex testing

Over the last decade, reflex testing, which involves the pathologist automatically performing biomarker tests based on the histopathology and origin of the tumor to determine gene alterations, has emerged as a strategy implemented in cancer centers to identify the biomarker status for a variety of cancers, positively impacting the accurate and prompt initiation of treatment and survival outcomes. This novel concept in clinical oncology is applied to melanoma, CRC, and various types of primary tumors. Implementing this method requires knowledge about which patients will benefit from biomarker testing and at what point in the disease course it is appropriate to test [1]. The use of reflex testing reduces the time to treatment initiation.

For example, biomarker testing is performed immediately after the pathological diagnosis of Non-small cell lung cancer (NSCLC), allowing patients to arrive at their clinical oncology appointment with their biomarker status. This can reduce the median treatment time by 21 days [27].

Melanoma

Internationally, reflex testing is indicated for advanced (stages III and IV) melanoma [28]. The pathologist should order the test immediately for early treatment initiation. It is indicated in thick tumors with a Breslow depth of 2-4 or >4 mm with or without ulceration and in all patients with nodal involvement (i.e., stage III) or lymphatic progression (satellitosis or in-transit metastasis) [1,5].

CRC

*BRAF* mutation testing is mainly indicated at diagnosis of mCRC since it may influence first-line therapy choice [29]. However, it may also be used in early CRC with MSI-H to exclude Lynch syndrome. Mutated *BRAF* has also been associated with MSI-H, mainly in tumors with hypermethylation of the MLH1 promoter and in right-sided colon cancers, mucinous histology, and serrated adenoma pathway [11,30]. In mCRC, *BRAF*-V600E testing can be performed either simultaneously with *RAS* testing or stepwise after excluding a *RAS* mutation. However, the simultaneous approach by applying focused NGS is recommended because it may provide information about non-V600E *BRAF* mutations [15].

*KRAS* and *BRAF* genes should be tested for mutations as reflex tests as soon as the histopathological diagnosis is reported either on the primary site or metastatic site tissues in mCRC cases before starting the first-line treatment. This usually occurs within an algorithmic protocol parallel to MSI testing using IHC or PCR-based techniques (e.g., Maxwell®, Promega Corporation, Madison, Wisconsin, United States; Idylla™, Biocartis Group, Mechelen, Belgium) [15].

Besides therapeutic purposes, it is helpful to perform *BRAF* mutational testing to differentiate between hereditary (e.g., Lynch Syndrome) and somatic CRC, especially when the neoplasia shows MSI with loss of MLH-1 expression shown by IHC and there is no ability to test MLH-1 methylation to confirm somatic origin. In such cases, the presence of *BRAF* mutation would clarify and confirm the somatic origin of CRC [15,16].

Gaps and barriers

The expert panel of oncologists and pathologists from Argentina, Brazil, Colombia, Costa Rica, and Mexico shared their experiences and polled colleagues to provide insight into current clinical practice for patients with *BRAF*-mutated melanoma and CRC. Together, they developed a list of perceived barriers to optimal care in their healthcare systems for early *BRAF* mutation screening and recommendations to remove the obstacles.

Argentina

*BRAF* testing is offered by the industry for metastatic melanoma and is available in several hospitals and clinics. On the other hand, *BRAF* testing for CRC is covered by a few private insurance companies and a couple of public oncology centers.

Brazil

Private health insurance in Brazil must cover *BRAF* and *MEK* inhibitors for patients with either resected high-risk or metastatic melanoma harboring *BRAF* V600E or V600K mutations. However, most health insurance companies do not cover *BRAF *testing. A pharmaceutical company has recently provided free *BRAF* testing. Unfortunately, *BRAF* inhibitors are not available in the public healthcare system. Therefore, only a few public institutions provide testing for melanoma patients for research purposes, and most rely on industry-sponsored *BRAF* testing.

On the other hand, public and private institutions routinely use industry-sponsored *K-RAS*, *N-RAS*, and *BRAF*-V600E testing for mCRC. However, BRAF inhibitors are still unavailable in public or private systems for mCRC. Out-of-pocket payment is typically needed if a patient wants to use a *BRAF* inhibitor for CRC. Therefore, the role of *RAS* and *BRAF* testing in CRC in many centers in Brazil is to appropriately select patients for anti-eGFR therapy in the first-line setting, since panitumumab or cetuximab is usually available in both public and private systems.

Colombia

The industry pays for the *BRAF* test (PCR) in cases with treatment indications in Colombia. Furthermore, according to the new resolution (2022), all tests in patients with patients are covered by the national healthcare system, which covers the *BRAF* test in cases with clinical indications. The test is performed mainly in reference laboratories. The test price is approximately $300 USD for PCR and $150 USD for IHC.

Costa Rica

Most melanoma and CRC cases in Costa Rica are treated in the public healthcare system (Caja Costarricense de Seguro Social). One of the main social security hospitals includes a molecular oncology laboratory where all cancer mutational analyses are run, using platforms such as NGS (Ion Torrent S5 (Thermo Fisher Scientific Inc., Waltham, Massachusetts, United States), oncomine focusing on 52 genes including* BRAF*) or other techniques like pyrosequencing or Idylla.

No private laboratory has the capability to identify *BRAF* mutations. Therefore, most patients are referred to social security hospitals, or their samples are sent to the United States for mutational analysis. Pharmaceutical companies, patients, or private insurers pay for outsourced testing. However, the public healthcare system does not cover any targeted treatment for *BRAF*-mutated tumors. Therefore, testing for this molecular alteration does not contribute to determining systemic therapy for affected patients with melanoma or CRC. However, pathological laboratories can analyze and interpret such results. Although some patients can access novel therapies through long legal processes, access to innovative therapies is still challenging.

Mexico

Although covered by industry, in 2021, only 88 *BRAF* mutation tests (mainly for melanoma) were performed, although there were 2051 people diagnosed with melanoma. No *BRAF* tests were performed for CRC. Although private insurance pays for some treatments, treatment options are lacking in the public healthcare system, so some providers do not test for the mutation [7].

Challenges to timely *BRAF* testing

Lack of Knowledge

Oncologists may not know enough about *BRAF* to choose the best oncologic treatment. Patients lack the information to advocate for themselves. More molecular biology education on targeted therapies with clinical applications is needed.

Lack of Treatment Options

The lack of novel drugs, like *BRAF*-targeted treatments, may contribute to high fatality rates in LA. Regulatory delays and/or costs are usually the issues. The latter is an issue in Brazil, where most of the population depends on a public healthcare system with limited resources. Notably, for melanoma, only 10% of the new medications introduced into the market in recent years have reached LA [31].

Lack of Equitable Distribution

Outsourcing predictive biomarker testing increases costs, turnaround times, and hospital workflow since information is distributed across electronic platforms. Oncologists and others cannot easily access reports. Third-party testing involves logistical challenges such as specimen handling and recovery by the referring center [32,33]. These difficulties hinder initial treatment.

Latin American researchers struggle to obtain reagents and equipment. Imported scientific supplies are highly taxed; hence research goods are significantly pricier than in HICs. Additionally, customs bureaucracy may delay merchandise delivery, which affects product quality. In addition, there is no free competition among reagent suppliers who have formed a monopoly. Finally, most Latin American countries concentrate their resources and research in large cities, which disadvantages smaller cities and hinders scientific collaboration.

Late Access to Biomarker Results

Because reflex testing is not widely available in LA, the turnaround time for biomarker results leads to potential treatment changes and delays. Many oncologists favor immunotherapy for melanoma initially, perhaps since it is readily accessible and precludes the need for *BRAF* testing. The same applies to mCRC, as most oncologists initiate chemotherapy before obtaining the molecular profile, adding the appropriate targeted agents once the test results are available.

Lack of Local Data on BRAF Mutations

Compared to North America and Europe, epidemiologic data and cancer registries are scarce in LA. Most are databases on cancer incidence and frequency. Despite the evident importance of *BRAF *testing in the diagnosis, treatment, and prognosis of melanoma and CRC, there is a paucity of molecular data. Several barriers impede the development of the genetic landscape in LA, undermining the potential for evolving therapies and personalized care.

First, *BRAF*-mutation screening requires genetic information from country-specific populations. LA's mix of people of Native American, European, and African descent potentially alters cancer patterns and undermines screening efficiency [34]. The scarcity of data is evident in Mexico, where the status and clinical relevance of *BRAF* mutation have not been thoroughly investigated [35]. Second, the lack of access to modern diagnostic tools like molecular testing in the region delays disease detection and diagnosis, hampering treatment efforts. Based on these data, an urgent requirement is to examine the gaps and barriers surrounding *BRAF *testing in LA.

Recommendations to address barriers

**Figure 2:**
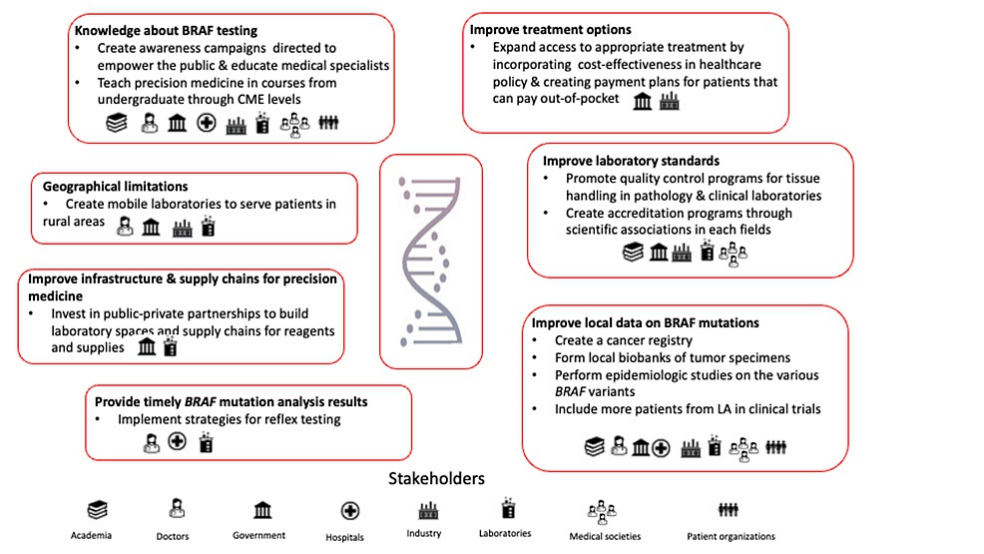
Recommendations and stakeholders responsible for implementation LA: Latin America; CME: continuing medical education Image Credit: Authors

Recognizing that LA is a diverse region with heterogeneous healthcare systems, we acknowledge that different countries and regions within countries have varying needs. Our recommendations are based on our experience in our healthcare systems and cannot reflect the needs of all people in LA. Figure 2 depicts the stakeholders responsible for each of the recommendations.

Lack of Knowledge

To combat the general lack of knowledge, governmental healthcare institutions should promote and support precision medicine, for example, through continuing medical education (CME) programs. Additionally, precision medicine awareness campaigns should be created. Knowledge can empower patients to advocate for access to appropriate tests and treatment. For example, being educated about *BRAF* mutations, how to test for them, and their impact on cancer treatment empowers patients to pressure their doctors and legislators to have access to the most appropriate therapies. NGOs could create social media campaigns. Informed patients are better equipped to advocate for themselves and discuss their options with healthcare providers during decision-making. Lastly, all members of the multidisciplinary team should be aware of the importance of *BRAF* testing. Information about when to test for *BRAF*, optimal testing techniques, and clinical impact must be widely spread and accepted among multidisciplinary teams.

Geographical Limitations

Fundamental changes in healthcare delivery are needed to close the precision medicine treatment gap between high-socioeconomic status (SES) urban patients and those in underserved locations. This can only be done by taking the means, expertise, and technologies to the areas where patients are.

Lack of Treatment Options

Access to appropriate and affordable treatment options for patients who test positive for the *BRAF* mutation should be expanded. Although guidelines recommend automatic *BRAF* testing for melanoma and CRC, only about half of the hospital clinics perform the test, possibly because healthcare providers know that treatment is unavailable. Cost-effective analysis in health policy should be incorporated, with an understanding that precision medicine has an upfront cost for better outcomes. Novel agreements regarding payments should also be explored. For example, reducing service fees or providing payment plans will allow more people to access testing and treatment. Additionally, the development of precision medicine education programs should be undertaken targeting undergraduate, graduate, residency, and CME programs for those responsible for the medical care of patients with cancer.

Lack of Infrastructure and Supply Chains

The development of public-private partnerships could help overcome the lack of laboratories available for testing and address the lack of regionally available supplies.

Lack of Quality Control in Laboratories

Promoting the implementation of quality control programs for pathology and clinical laboratories, as well as accreditation support programs, through scientific associations in each field of specialty could lead to increased overall monitored quality in laboratories. Creating guidelines and quality control protocols for tissue handling in the pre-analytical phase should be mandatory. Further, surgeons, dermatologists, and endoscopists must be trained to handle specimens properly and follow guidelines. Quality control is necessary to ensure that samples are treated appropriately.

Late Access to Biomarker Results

Strategies for reflex testing of all biomarkers in all tumors with clinically actionable mutation profiles, including *BRAF*, should be implemented to enable early therapy initiation for patients with private insurance or the means to pay out-of-pocket.

Lack of Local Data on BRAF Mutations

Create a registry to capture real-world data on *BRAF*-V600+ melanoma and CRC. Some countries have begun collecting epidemiological data, but more than incidence is needed. Cancer registries help identify the burden and design cancer-control efforts. Registries that collect all test results and real-world data will allow researchers to implement precision medicine fully. Collecting data from LA is essential because most of the epidemiologic data come from Europe and the United States and might not represent all people in LA. Governmental programs and private initiatives should support epidemiologic research programs involving public/private partnerships, governmental and non-governmental organizations (NGOs), and patient associations. In addition, more patients from the LA region should be included in clinical trials to explore the effect of targeted therapies in this population. Lastly, medical communities should collaborate to create local tumor specimen biobanks.

## Conclusions

There is an urgent need for effective treatment and greater access to personalized care in melanoma and CRC in LA that would reduce the substantial burden of these diseases in the region. Needs assessment projects such as the one in this article must be carried out to ensure that public health officials, health care providers, and drug regulatory agencies are aware of the existing barriers that affect patients, their families, and society. Simple actions can move the region toward equitable precision medicine, and all stakeholders are needed.
