# Correction: BRAF Testing in Melanoma and Colorectal Cancer in Latin America: Challenges and Opportunities

**DOI:** 10.7759/cureus.c83

**Published:** 2022-12-02

**Authors:** Renata D Peixoto, Jad Joseph Abbas Chakhtoura, José L Aguilar-Ponce, Hernan Garcia-Rivello, Angela M Jansen, Rafael Parra Medina, Allan Ramos-Esquivel, Stephen Doral Stefani

**Affiliations:** 1 Medical Oncology, Centro Paulista de Oncologia, Sao Paulo, BRA; 2 Anatomical Pathology, Hospital Rafael Angel Calderón Guardia, San Jose, CRI; 3 Oncological Center, Médica Sur, Mexico City, MEX; 4 Pathology, Hospital Italiano de Buenos Aires, Buenos Aires, ARG; 5 Pathology, Institute of Translational Medicine and Biomedical Engineering (IMTIB), Buenos Aires, ARG; 6 Genetics, Americas Health Foundation, Laguna Beach, USA; 7 Pathology, Instituto Nacional de Cancerología, Bogota, COL; 8 Caja Costarricense de Seguro Social, Hospital San Juan de Dios, San José, CRI; 9 Health Technology Assessment, Unimed, Porto Alegre, BRA

This article has been corrected to include two authors who were erroneously omitted from the author list by the submitting author. The following authors have been added in 3rd and 7th position, respectively:

- José L. Aguilar-Ponce Oncological Center, Médica Sur, Mexico City, MEX
 
- Allan Ramos-Esquivel Caja Costarricense de Seguro Social, Hospital San Juan de Dios, San José, CRI

The submitting author sincerely apologizes for the oversight.

